# Antioxidant and pro-oxidant effects of oil palm (*Elaeis guineensis*) leaves extract in experimental diabetic nephropathy: a duration-dependent outcome

**DOI:** 10.1186/1472-6882-13-242

**Published:** 2013-09-29

**Authors:** Rajavel Varatharajan, Munavvar Zubaid Abdul Sattar, Ivy Chung, Mahmood Ameen Abdulla, Normadiah M Kassim, Nor Azizan Abdullah

**Affiliations:** 1 Department of Pharmacology, Faculty of Medicine, University of Malaya, 50603 Kuala Lumpur Malaysia; 2 School of Pharmaceutical Sciences, University Sains Malaysia, 11800 Penang, Malaysia; 3 Department of Molecular Medicine, Faculty of Medicine, University of Malaya, 50603 Kuala Lumpur, Malaysia; 4 Department of Anatomy, Faculty of Medicine, University of Malaya, 50603 Kuala Lumpur, Malaysia

**Keywords:** Diabetic nephropathy, Oil palm leaves extract, Kidney, Oxidative stress

## Abstract

**Background:**

Catechins-rich oil palm (*Elaeis guineensis*) leaves extract (OPLE) is known to have antioxidant activity. Several polyphenolic compounds reported as antioxidants such as quercetin, catechins and gallic acid have been highlighted to have pro-oxidant activity at high doses. Therefore, the present study was conducted to investigate the antioxidant and pro-oxidant effects of chronically administering high dose of OPLE (1000 mg kg^-1^) in an animal model of diabetic nephropathy (DN).

**Methods:**

Animal body weight, indexes of glycaemia, renal function and morphology were assessed in diabetic animals with and without OPLE (1000 mg kg^-1^) for 4 and 12 weeks respectively. Oxidative stress was quantified by measuring levels of 8-hydroxy-2’-deoxyguanosine (8-OHdG), lipid peroxides (LPO) and reduced glutathione (GSH). Transforming growth factor-beta1 (TGF-β1), a key mediator of extracellular matrix accumulation, was analysed in plasma. The mechanisms of OPLE action were evaluated by assessing nicotinamide adenine dinucleotide phosphate (NADPH) oxidase subunits (p22phox and p67phox) expression.

**Results:**

Oral administration with high dose of catechins-rich OPLE (1000 mg kg^-1^) to STZ-induced diabetic rats for 4 weeks attenuated renal dysfunction (hyperfiltration, proteinuria) and development of glomerulosclerosis and tubulointerstitial fibrosis, features that are associated with DN. Suppression of increases in oxidative stress markers (8-OHdG, LPO) and the fibrotic cytokine, TGF-β1 was observed. OPLE also reduced renal expression of NADPH oxidase subunits p22phox and p67phox. In contrast and surprisingly, identical dose of OPLE when administered to diabetic animals for 12 weeks caused worsening of renal dysfunction, histopathology in addition to further elevation of oxidative stress marker (LPO) and TGF-β1. These unfavourable effects of prolonged treatment with 1000 mg kg^-1^ OPLE were accompanied by increase expression of one of the NADPH oxidase subunits, p22phox.

**Conclusion:**

Our study indicates that chronic administration of 1000 mg kg^-1^ OPLE exerts both antioxidant and pro-oxidant effects in DN depending on the duration of treatment. The present study also reveals that the antioxidant/pro-oxidant effects of OPLE are in part, due to modulation of NADPH activity.

## Background

The balance between the production of reactive oxygen species (ROS), notably superoxide anion (O_2_^-^) and hydrogen peroxide (H_2_O_2_), and the antioxidant defence system that includes superoxide dismutase (SOD) and glutathione (GSH), is believed to be vital in maintaining healthy biological systems. Indeed the balance between oxidation and anti-oxidation which determines the degree of oxidative stress is disrupted by disease state. Experimental and clinical evidence indicates that oxidative stress may contribute to the initiation and development of diabetic nephropathy (DN) [1-4] and in support, previous study in our laboratory also provided evidence for *in vivo* oxidative stress in kidney of diabetic rats that was accompanied by renal dysfunction such as glomerular hyperfiltration and proteinuria; and structural damage that included glomerulosclerosis and tubulointerstitial fibrosis [5]. In the diabetic state, chronic hyperglycaemia can activate multiple pathways that lead to increased generation of O_2_^-^ and other ROS; some of these pathways include enhanced activity of the mitochondrial electron transport chain [2], increased expression and uncoupling of endothelial nitric oxide synthase [6] and activation of the reduced forms of nicotinamide adenine dinucleotide phosphate (NADPH) [7,8]. The latter system is present abundantly in the renal vessels and in the glomerular mesangial and podocyte cells, the macula densa, and the thick ascending limb, distal tubule, and collecting ducts [7]. Moreover, the renal expression of NADPH oxidase has been shown to be enhanced in an animal model of DN [9].

Strategies that reduce oxidative stress and/or increase the activity of antioxidant defence mechanism can therefore attenuate hyperglycaemia-induced renal injury such as in DN. Oil palm (*Elaeis guineensis*) leaves extract (OPLE) is rich in catechins and these polyphenolic compounds are considered to have antioxidant activity that is several folds higher than that of vitamins C and E [10]. We have demonstrated that oral administration of OPLE (200 mg kg^-1^, 500 mg kg^-1^ administered daily over a period of 4 and 12 weeks respectively) improved renal function and prevented kidney structural injury in rats with streptozotocin-induced diabetes. We ascribed the beneficial effects of OPLE in DN to its antioxidant effect given that OPLE suppressed the elevation of oxidative stress markers and improved antioxidant defences [5].

Although the antioxidant capacity of phytochemicals is well recognised [11,12], they can also in contrast, display pro-oxidant activities under certain conditions, such as at high doses or in the presence of metal ions. For certain, the pro-oxidant or antioxidant activity very much depends on their concentration. Several polyphenols previously known as antioxidants such as quercetin, catechins and gallic acid have been highlighted to have pro-oxidant activity at high doses [13-16]. For instance, the antioxidant activity of quercetin was observed only at low doses (0.1 – 20 μM) while higher doses (50 μM) potentiated O_2_^-^ generation within isolated mitochondria and cultured cells [14,15]. It is with this knowledge that prompted us to investigate whether high concentration of OPLE (1000 mg kg^-1^) administered over a period of 4 and 12 weeks respectively to rats with streptozotocin-induced diabetes, nonetheless maintains its antioxidant effect in DN in the same way as in our previous study which utilised lower doses of the extract (200 mg kg^-1^ and 500 mg kg^-1^ respectively). On the other hand, we do not exclude the possibility that high dose of OPLE, depending on the duration of treatment, may perhaps aggravate oxidative stress in our animal model of DN. To test the above hypothesis, we assess the effect of treatment with 1000 mg kg^-1^ of OPLE on animal body weights, indexes of glycaemia, renal function and morphology. We establish the extent of oxidative stress and the modulation of this parameter by OPLE by measuring levels of 8-hydroxy-2’-deoxyguanosine (8-OHdG), lipid peroxides (LPO) and GSH. Further, we elucidate the mechanisms by which OPLE exert its antioxidant effect or its aggravation of oxidative stress in DN.

## Methods

### Chemicals

Standardised ethanol extract of oil palm leaf extract (OPLE) was obtained from Nova Laboratories, Malaysia (Batch no: WH 1446). Antibodies recognising NADPH oxidase subunit p22phox and β-actin were purchased from Santa Cruz Biotechnology (USA). Antibodies recognising NADPH oxidase subunit p67phox was procured from Gene Tex (USA). Enzyme-linked immunosorbent assay (ELISA) for the determination of glutathione (GSH), lipid peroxides (LPO) and 8-hydroxy-2-deoxy guanosine (8-OHdG) were obtained from Cayman Chemicals (Ann Arbor, MI, USA). ELISA kit for the determination of transforming growth factor-beta 1 (TGF-β1) was obtained from Abnova (Walnut, USA). Streptozotocin (STZ) was purchased from Sigma-Aldrich (St. Louis, MO, USA). Immunohistochemical stains were obtained from Dako (Baltimore, MD, USA). All other chemicals and solvents were of analytical grade and were obtained from commercial suppliers in Malaysia.

### Preparation of OPLE

Ethanolic fraction of oil palm leaves was prepared by the manufacturers as described in our previous study [5]. According to the manufacturer report, 1.1% (−) catechin gallate and 1.5% ferulic acid were present in the powder extract.

### General preparation of experimental animals

All experimental procedures were approved and complied with the Guidelines of the Care and Use of Laboratory Animals at University of Malaya in Kuala Lumpur (FAR/20101106/NAA-R). Male Sprague–Dawley rats weighing 270–330 g were acclimatized (under controlled conditions of 12 h light : dark cycle and at 21-25°C) for 1 week before the study and the rats were allowed unrestricted access to standard rat pellets and tap water during the entire study period. Animals were randomly divided into three groups for the 4-week or 12-week studies (n = 10-15 per group) namely control (C), diabetes control group (DC), diabetes group treated with OPLE at a dose of 1000 mg kg^−1^ (D + OPLE 1000). A single intraperitoneal (*i*.*p*.) injection of STZ at a dose of 60 mg kg^−1^ was administered to induce diabetes which was dissolved in freshly prepared 0.9% sodium chloride (pH 4.5). Control rats received an equal volume of 0.9% sodium chloride by *i*.*p*. injection. Diabetes was confirmed after 72 h by measuring blood glucose levels with the use of glucose oxidase reagent strips (one touch glucometer, Accu Chek) and those rats with a random blood glucose level of >12 mmol l^−1^ were selected for the study. After confirmation of diabetes, the rats were treated with 1000 mg kg^−1^ OPLE which was dissolved in distilled water given daily for either 4 or 12 weeks by oral administration. Age matched control and diabetic control group rats did not receive any OPLE treatment for the 4-week or 12-week study. Blood glucose level was measured in all groups twice in a week. On the 4^th^ week, the percentage of survivors amongst the C, DC and D + OPLE 1000 mg kg^−1^ were 100%, 80% and 90% respectively. On the 12^th^ week, the percentage of survivors amongst the C, DC and D + OPLE 1000 mg kg^−1^ were 100%, 70% and 75% respectively. At the end of the 4-week or 12-week periods, the rats were anaesthetised with sodium pentobarbital (60 mg kg^−1^, *i*.*p*.) for the renal functional study.

### Surgical preparation and functional studies

A tracheostomy was performed to facilitate respiration. The left jugular vein and carotid artery were cannulated for anaesthetic infusion (12.5 mg kg^−1^ h^−1^ at 3 ml h^−1^ in 140 mM NaCl) and arterial pressure recordings (Power Lab Systems and pressure transducer, AD Instruments) respectively while the left femoral artery was cannulated for blood sample collection. The left kidney was exposed via a flank incision and a catheter was placed in the urinary bladder for urine collection. An electromagnetic flow probe was placed on the renal artery for renal blood flow (RBF) measurement (Carolina Square-wave Electromagnetic flowmeter and EP 100 series probe; Carolina Medical, NC, USA). Inulin (10 mg ml^−1^) was included in the infusate. Thereafter, a 2 ml priming dose solution (10 mg ml^−1^ inulin in 140 mM NaCl) was administered to the rats via the jugular vein and allowed an hour as equilibration period. Thereafter, four 30 min clearances (urine) were collected and frozen at −20°C till subsequent analysis. Arterial blood samples were collected at the beginning and at the end of every two clearances. The blood samples were centrifuged and plasma was frozen at −20°C till subsequent analysis. At the end of the study, the rat was sacrificed by rapid intravenous injection of 1 ml sodium pentobarbital (60 mg kg^−1^) and the right kidney was harvested, dissected into cortex and medulla, and snap frozen in liquid nitrogen for measurement of GSH, LPO, NADPH oxidase subunits p22phox and p67phox. The left kidney was perfused first with ice-cold phosphate buffered saline until cleared off blood and then fixed with 10% formalin for histopathological screening.

### Biochemical analysis

Urinary protein concentration was determined by biuret reagent according to the method developed by Doumas et al. [17]. Urine and plasma sodium concentrations were analysed by using a flame photometer (Sherwood, Model 420, UK) and urine volume was measured gravimetrically. Inulin content in urine and plasma samples were determined according to the method developed by Somogyi et al. [18] after the samples were deproteinised according to the method proposed by Bojesen et al. [19]. Glomerular filtration rate (GFR) was equated with renal clearance of inulin and was expressed as ml min^−1^ g^−1^ of kidney weight.

### Urinary excretion of 8-OHdG

8-OHdG is one of the most common markers for oxidative DNA damage and oxidative stress *in vivo*. ELISA kit from Cayman Chemicals was used to measure 8-OHdG levels in 2 h urine samples. The 8-OHdG standards (0.5-80 ng ml^−1^) and 35–50 μl of urine were allowed to incubate separately for 1 h in a microtiter plate precoated with 8-OHdG. After washing the antibodies, enzyme-labelled secondary antibody was added and incubated for 1 h followed by washing. The colour that developed by the addition of 3, 3’, 5, 5’–tetramethylbenzidine was measured at 450 nm using a spectrophotometer. Urinary 8-OHdG was expressed as total amount excreted in 2 h.

### Reduced glutathione in kidney

Cytosolic reduced GSH in renal cortex homogenates was measured using a glutathione assay kit from Cayman Chemicals according to the manufacturer’s protocol. The sample (100–150 μg) was deproteinised using metaphosphoric acid, and the intensity of the yellow colour produced by 5-thio-2-nitrobenzoic acid in the supernatant liquid was measured at 410 nm using spectrophotometer (UV/VIS λ, Perkin Elmer, USA).

### Lipid peroxides in kidney

Lipid peroxides (LPOs) in the renal cortex were measured colourimetrically at 500 nm using Cayman’s assay kit. Briefly, renal cortex was homogenised in HPLC-grade water and LPOs were extracted from the homogenates according to the manufacturer’s protocol. LPO were measured directly by redox reactions with ferrous ions using the kit, and the resulting ferric ions were detected using thiocyanate ion as the chromogen.

### Plasma transforming growth factor-beta 1 (TGF-β1)

The frozen plasma samples collected from the arterial blood of experimental animals on the 4^th^ and 12^th^ week were analysed for TGF-β1 using ELISA kit as previously described [5]. Plasma TGF-β1 is expressed as total amount excreted in *pg* ml^−1^.

### Histopathological study

Tissue samples were collected at necropsy. After formalin fixation, renal tissues were processed using an automated tissue processing machine and finally embedded in paraffin. Subsequently, tissue sections were cut at 5 μm thickness using a microtome, dewaxed and stained with haematoxylin and eosin (H&E), periodic acid-Schiff (PAS) and Masson’s trichrome stains. Renal morphology changes within the glomeruli and interstitial areas were assessed with the aid of a Nikon Eclipse 80i light microscope, using a semi quantitative scoring method [20,21].

### Immunohistochemistry

Renal tissue was sectioned into 5 μm thickness using a rotary microtome and placed onto poly-L-lysine coated slides. For antigen retrieval, specimen slides were transferred to 10 mmol l^−1^ citrate buffer solution (pH 6.0) and then heated in decloaking chamber at 120°C for 20 min. Subsequently, the sections were incubated with Dako Real™ Peroxidase blocking solution for 10 min and rinsed with phosphate buffer saline (PBS) (pH 7.4). The sections were incubated with primary antibodies recognising p22phox (1:200) and p67phox (1:100) for 1 h at room temperature. The sections were rinsed with PBS (pH 7.4) and were incubated with horseradish peroxidase (HRP) rabbit/mouse secondary antibody (Dako Real™ Envision™) for 30 min at room temperature. For coloration, the slides were incubated with a mixture of Dako Real™DAB Chromogen and Dako Red™ substrate buffer (1:50) for 5 min at room temperature. Sections were finally counterstained with hematoxylin. Representative areas of renal morphology changes within the glomeruli and interstitial areas were photographed using a Nikon Eclipse 80i light microscope.

### Western blotting

Homogenised samples from the renal cortex were separated on 4-20% sodium dodecyl sulphate (SDS-PAGE) gels and the proteins were transferred to polyvinylidene fluoride (PVDF) membrane. The membranes were blocked with 5% non-fat milk followed by primary antibodies recognising p22phox and p67phox (1:500), and incubated at 4°C overnight. The membranes were washed and incubated with HRP-conjugated goat antirabbit IgG. Band densities were normalised to the total amount of protein loaded in each well, as determined by densitometric analysis of PVDF membranes stained with Amersham ECL Prime Western Blotting Detection Reagent (GE Healthcare). The proteins were visualised by chemiluminescence (UVP, Bio Spectrum, USA) and the densities of specific bands were quantitated by densitometry using Vision Work LS software (Version: 7.1 RC3.10). Housekeeping protein β-actin (1:1000) was used as loading control.

### Statistical analysis

Data are shown as mean ± SEM. The mean values were compared among the 3 groups using one way analysis of variance (ANOVA), followed by Tukeys Multiple Comparison Test (Graph Pad Prism). Experimental differences were considered statistically significant if *P* < 0.05.

## Results

### Effect of OPLE on metabolic parameters

The effects of OPLE on body and kidney weights, random blood glucose level and mean arterial pressure in C, DC and D + OPLE 1000 mg kg^−1^ are shown in Table 1. The mean body weight of DC group was 1.2 and 1.8 fold lower than the C group on the 4^th^ and 12^th^ week after diabetes induction respectively; however the difference was significant (*P* < 0.01) only in the 12-week study. In contrast, kidney weight normalised by 100 g body weight of the DC group was significantly heavier than that of the corresponding C group by 1.4 fold (*P* < 0.01) on the 4^th^ week. Similar result was obtained with DC group as compared to the corresponding C group on the 12^th^ week (2.2 fold, *P* < 0.001). However, diabetic rats treated with 1000 mg kg^−1^ OPLE did not show any significant changes in mean body weight and kidney to body weight ratio for both the 4-week and 12-week experimental studies.

**Table 1:** Effects of OPLE on body and kidney weights, random blood glucose level and mean arterial pressure

	**Control**		**Diabetes control**		**Diabetes + OPLE 1000 mg kg**^**-1**^	
	**4 weeks**	**12 weeks**	**4 weeks**	**12 weeks**	**4 weeks**	**12 weeks**
Body weight (g)	365.0 ± 14.3	428.3 ± 13.0	309.2 ± 25.4	235.0 ± 14.6^aaa^	291.7 ± 32.8	266.7 ± 15.2^aaa^
Kidney weight (g 100 g^-1^ body weight)	0.40 ± 0.01	0.32 ± 0.02	0.57 ± 0.03^aa^	0.69 ± 0.02^aaa^	0.56 ± 0.04^aa^	0.64 ± 0.03^aaa^
Glucose level (mmol l^-1^)	4.7 ± 0.1	6.3 ± 0.2	30.7 ± 1.7^aaa^	31.0 ± 1.2^aaa^	27.0 ± 2.3^aaa^	31.7 ± 0.8^aaa^
Mean arterial pressure (mmHg)	113.2 ± 2.1	111.5 ± 2.9	115.3 ± 3.2	116.7 ± 3.3	115.0 ± 2.8	114.2 ± 2.5

Data are expressed as mean ± SEM of six experiments for each group. ^*aa*^*P* < 0.01; ^*aaa*^*P* < 0.001 vs. corresponding control.

Random glucose levels in the whole blood of DC rats were significantly higher than that of the C rats at both 4 (6.6 fold, *P* < 0.001) and 12 weeks (5 fold, *P* < 0.001) after diabetes induction. OPLE (1000 mg kg^−1^) treatment of diabetic animals for either 4 or 12 weeks did not show any improvement in the random glucose level (Table 1).

### Effect of OPLE on renal function in diabetic rats

Renal blood flow (RBF) of various groups in the present study is listed in Table 2. RBF did not differ among the three study groups at 4 weeks. Conversely, RBF was significantly increased in DC group compared with the corresponding C group at 12 weeks (1.5-fold, *P* < 0.001) and OPLE at 1000 mg kg^−1^ did not normalise the RBF in the diabetic group.

**Table 2:** Effect of OPLE on renal functional parameters

	**Control**		**Diabetes control**		**Diabetes + OPLE 1000 mg kg**^**-1**^	
	**4 weeks**	**12 weeks**	**4 weeks**	**12 weeks**	**4 weeks**	**12 weeks**
Renal blood flow (ml min^-1^ g^-1^ kidney)	1.43 ± 0.10	1.66 ± 0.15	2.28 ± 0.39	2.49 ± 0.17^aaa^	1.73 ± 0.32	2.52 ± 0.19^aaa^
Glomerular filtration rate (ml min^-1^ g^-1^ kidney)	0.61 ± 0.06	0.47 ± 0.06	0.96 ± 0.04^aaa^	0.80 ± 0.05^a^	0.62 ± 0.06^bb^	1.06 ± 0.14^aaa,b^
Urine flow rate (μl min^-1^ g^-1^ kidney)	3.75 ± 0.59	2.72 ± 0.36	10.68 ± 1.30^aaa^	7.32 ± 0.57^aaa^	5.40 ± 0.68^bb^	10.88 ± 0.98^aaa,bb^
Urinary protein excretion (mg 2hr^-1^)	1.33 ± 0.03	1.08 ± 0.02	6.83 ± 0.30^aaa^	3.72 ± 0.30^aa^	1.77 ± 0.17^bbb^	5.99 ± 0.44^aaa,bb^
Fractional sodium excretion (%)	0.63 ± 0.19	0.50 ± 0.08	1.34 ± 0.34	0.91 ± 0.20	1.77 ± 0.17^bbb^	5.99 ± 0.44^aaa,bb^

Data are expressed as mean ± SEM of six experiments for each group. ^*a*^*P* < 0.05; ^*aa*^*P* < 0.01; ^*aaa*^*P* < 0.001 vs. corresponding control; ^*b*^*P* < 0.05, ^*bb*^*P* < 0.01, ^*bbb*^*P* < 0.001 vs. corresponding diabetes control.

It is well established that early stages of DN are associated with hyperfiltration, both clinically and experimentally. Glomerular filtration rate (GFR) in the 4-week DC group was raised in comparison with the corresponding C group (1.6-fold, *P* < 0.001), as evaluated by inulin clearance (Table 2). Hyperfiltration was prevented by 1000 mg kg^−1^ OPLE (*P* < 0.01) in the 4-week study.

Likewise, GFR was significantly greater (1.7-fold, *P* < 0.05) in the 12-week DC rats than in the corresponding C rats. But OPLE treatment at 1000 mg kg^−1^ of diabetic animals for 12 weeks further increased the GFR (*P* < 0.05), signifying further renal dysfunction.

### Renal excretory function

The urine flow rate (UFR) of the DC rats was remarkably increased in comparison to C rats on both the 4^th^ week (2.8 fold, *P* < 0.001) and 12^th^ week (2.7 fold, *P* < 0.001) and this occurrence may be the outcome of hyperfiltration (Table 2). OPLE at 1000 mg kg^−1^ administered over a period of 4 weeks almost prevented the increase in UFR in diabetic rats (*P* < 0.01). However, when the same dose of OPLE was administered over a period of 12 weeks to diabetic animals, UFR was further increased (*P* < 0.01).

Protein excretion in the urine indicates damage to glomerular barrier, thus urinary protein excretion was measured in the three experimental groups (Table 2). Urinary protein excretion was increased significantly in DC rats compared with C rats in both the 4-week (5-fold, *P* < 0.001) and 12-week (3.4-fold, *P* < 0.01) studies. Proteinuria induced by diabetes was significantly reduced by 1000 mg kg^−1^ OPLE administered over a period of 4 weeks (*P* < 0.001). On the contrary, when the extract was administered for 12 weeks to diabetic animals, proteinuria was aggravated (*P* < 0.01), indicating more renal damage.

Fractional sodium excretion was evaluated to assess tubular function. However, our study did not show any differences amongst the three experimental groups in both the 4-week and 12-week studies (Table 2).

### Effect of OPLE on oxidative stress

Oxidative stress was assessed by estimating the formation of oxidative damage products or endogenous end-products of ROS in plasma, urine and renal cortex of the STZ-diabetes induced rats to address the effect of OPLE on oxidative stress.

### 8-Hydroxy-2-deoxy guanosine (8-OHdG)

**Figure 1:**
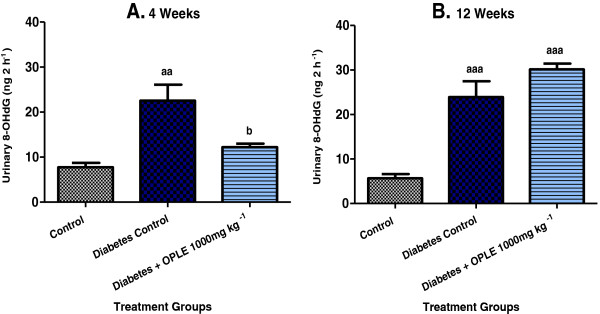
**Effect of OPLE on urinary 8**-**OHdG concentration in the 4-week (A) or 12-week (B) study.** Data are expressed as mean ± SEM (*n* = 6 per group). ^*aa*^*P* < 0.01; ^*aaa*^*P* < 0.001 vs. corresponding control; ^*b*^*P* < 0.05 vs. corresponding diabetes control.

In this study, we examined the oxidative DNA damage in kidneys of STZ-induced diabetic rats by measuring the levels of 8-OHdG in urine samples. Urinary 8-OHdG levels were significantly greater in DC rats than in C rats on the 4^th^ week (22.56 ± 3.51 ng 2 h^−1^ vs. 7.77 ± 0.93 ng 2 h^−1^, *P* < 0.01) and the 12^th^ week (23.91 ± 3.58 ng 2 h^−1^ vs. 5.68 ± 0.95 ng 2 h^−1^, *P* < 0.001) after diabetes induction as shown in Figure 1. Administration of 1000 mg kg^−1^ OPLE to diabetic rats for 4 weeks significantly reduced urinary 8-OHdG levels (12.23 ± 0.75 ng 2 h^−1^, *P* < 0.05) when compared to DC rats. In contrast, 1000 mg kg^−1^ OPLE given to diabetic rats for 12 weeks further raised the urinary 8-OHdG level (30.14 ± 0.75 ng 2 h^−1^) as compared to DC rats; however the difference is not significant.

### Lipid peroxides (LPO)

**Figure 2:**
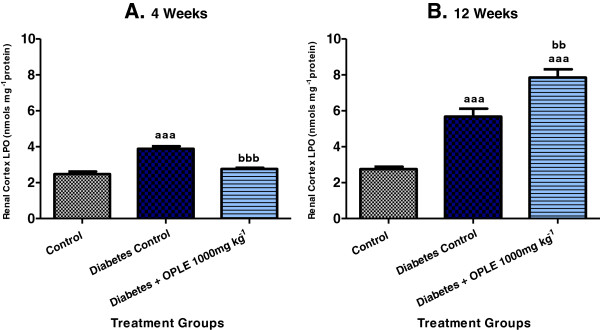
**Effect of OPLE on renal cortical LPO concentration in the 4-week (A) or 12-week (B) study.** Data are expressed as mean ± SEM (*n* = 6 per group). ^*aaa*^*P* < 0.001 vs. corresponding control; ^*bb*^*P* < 0.01, ^*bbb*^*P* < 0.001 vs. corresponding diabetes control.

Renal cortical LPO were significantly higher in DC rats than in C rats at both 4 weeks (3.88 ± 0.15 nmols mg^−1^ protein vs. 2.48 ± 0.14 nmols mg^−1^ protein, *P* < 0.001) and 12 weeks (5.68 ± 0.44 nmols mg^−1^ protein vs. 2.75 ± 0.14 nmols mg^−1^ protein, *P* < 0.001) after diabetes induction as shown in Figure 2. Significant reduction of renal cortical LPO levels was observed in the D + OPLE 1000 mg kg^−1^ group (2.76 ± 0.07 nmols mg^−1^ protein, *P* < 0.001) in the 4-week study but in the 12-week study, the D + OPLE 1000 mg kg^−1^ rats showed further increase in LPO levels (7.86 ± 0.46 nmols mg^−1^ protein, *P* < 0.001) when compared to DC rats; indicating that prolonged administration of 1000 mg kg^-1^ OPLE over a period of 12 weeks aggravate renal oxidative stress.

### Glutathione (GSH)

**Figure 3:**
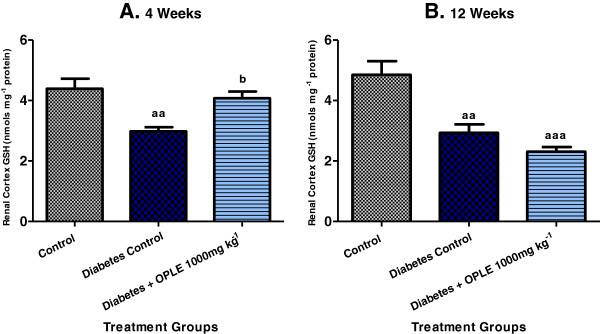
**Effect of OPLE on kidney (renal cortex) GSH concentration in the 4-week (A) or 12-week (B) study.** Data are expressed as mean ± SEM (*n* = 6 per group). ^*aa*^*P* < 0.01, ^*aaa*^*P* < 0.001; vs. corresponding control; ^*b*^*P* < 0.05 vs. corresponding diabetes control.

GSH is a component of the endogenous antioxidant defence system and it plays a major role in scavenging hydrogen peroxide (H_2_O_2_) under physiological conditions. The measurement of renal GSH content was performed to establish the effect of OPLE on endogenous antioxidant defence system in diabetes. As demonstrated in Figure 3, the reduction in renal cortical GSH content was significantly improved by 1000 mg kg^−1^ OPLE in comparison to DC rats on the 4^th^ week (4.08 ± 0.22 nmols mg^−1^ protein vs. 2.98 ± 0.13 nmols mg^−1^ protein, *P* < 0.05). But when 1000 mg kg^−1^ OPLE was administered to diabetic rats for 12 weeks, there was further reduction, albeit not significant, of renal GSH (2.30 ± 0.15 nmols mg^−1^ protein vs. 2.93 ± 0.28 nmols mg^−1^ renal GSH in DC rats).

### Effect of OPLE on plasma transforming growth factor beta-1 (TGF- β1)

**Figure 4:**
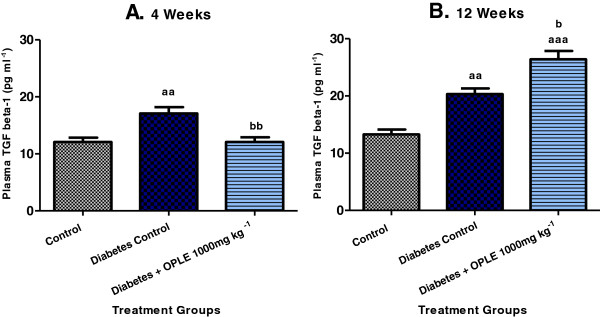
**Effect of OPLE on plasma TGF-β1 concentration in the 4-week (A) or 12-week (B) study.** Data are expressed as mean ± SEM (*n* = 6 per group). ^*aa*^*P* < 0.01; ^*aaa*^*P* < 0.001 vs. corresponding control; ^*b*^*P* < 0.05, ^*bb*^*P* < 0.01 vs. corresponding diabetes control.

In accordance with renal dysfunction and the increased markers of oxidative stress (urinary 8-OHdG excretion and renal cortical LPO), significantly higher concentrations of TGF-β1 were detected in plasma of DC rats than in C rats on both the 4^th^ week (17.09 ± 1.10 pg ml^−1^ vs. 12.09 ± 0.75 pg ml^−1^, *P* < 0.01) and 12^th^ week (20.34 ± 0.96 pg ml^−1^ vs. 13.29 ± 0.85 pg ml^−1^, *P* < 0.01) after diabetes induction, as shown in Figure 4. When 1000 mg kg^−1^ OPLE was administered to diabetic animals for 4 weeks, a significant reduction in plasma TGF-β1 concentrations was observed as compared to DC rats (12.09 ± 0.82 pg ml^−1^, *P* < 0.01). On the other hand, when the extract was administered for an extended period i.e. 12 weeks to diabetic animals, a further increase in TGF-β1 concentrations was observed in comparison to DC rats (26.42 ± 1.4 pg ml^−1^, *P* < 0.01); indicating that more fibrotic changes within the kidney might have occurred.

### Effect of OPLE on renal morphology

**Figure 5:**
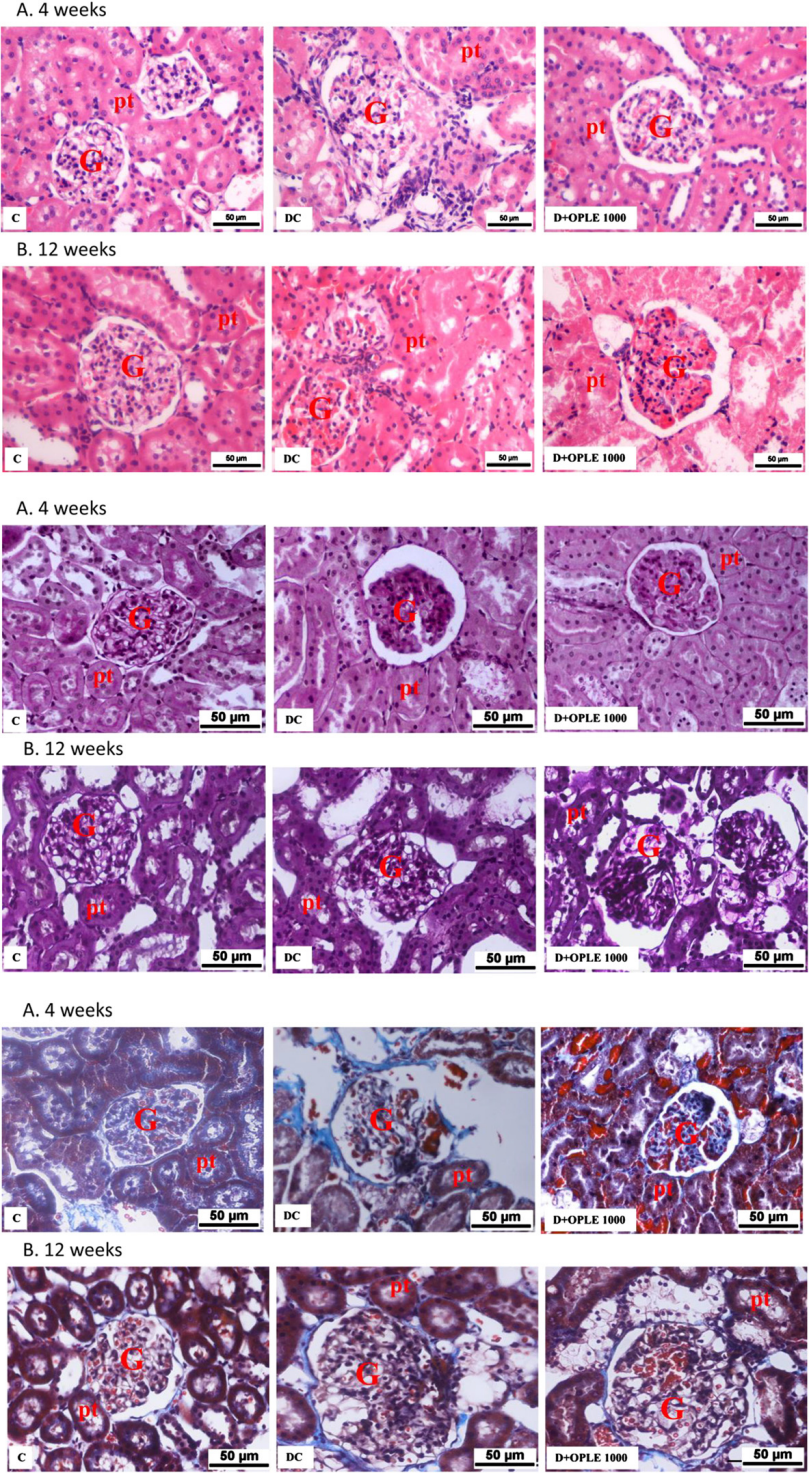
**Effect of OPLE on renal morphology**. Histological sections of kidneys stained with (i) haematoxylin and eosin; (ii). Periodic acid-Schiff (PAS); (iii). Masson’s trichrome in the 4-week **(A)** or 12-week **(B)** study. Control (C), Diabetes Control (DC), Diabetes + OPLE 1000 mg kg−1 (D+OPLE 1000); G, glomerulus; pt, proximal tubule; Bar = 50 μm.

The photomicrographs of haematoxylin and eosin (H&E) staining of renal tissues of control and experimental groups of rats are shown in Figure 5i(A, B). Renal tissue sections of C rats demonstrated normal architecture with normal glomeruli and tubules. Renal sections from DC rats exhibited glomerular injury, tubular vacuolization-necrosis, interstitial oedema and interstitial infiltration by inflammatory cells. Very minimal histological changes were detected in kidney of diabetic animals treated 1000 mg kg^-1^ OPLE for 4 weeks and the sections were comparable to C rats. Diabetic animals treated with the extract for 12 weeks however revealed more severe damage than the DC rats.

PAS-stained sections of the renal cortex 4 weeks after diabetes induction (Figure 5iiA) exhibited marked glomerulosclerosis, characterized by glomerular basement membrane thickening and mesangial expansion with glomerular hypertrophy, in comparison with C rats. Remarkably, treatment with 1000 mg kg^-1^OPLE for 4 weeks reduced glomerulosclerosis and attenuated the mesangial matrix accumulation in the diabetic rats. Morphometric analysis revealed a significant reduction in the mesangial area in the D + OPLE 1000 mg kg^−1^ rats and the data clearly demonstrates that OPLE treatment alleviated the mesangial expansion (*P* < 0.05, Table 3). Twelve weeks of oral administration of the extract however did not resolve the diabetes-induced renal damage, if anything there was worsening of renal injury, albeit not significant in comparison to DC rats (Figure 5iiB, Table 3).

**Table 3:** Effects of OPLE on renal structure

	**Control**		**Diabetes control**		**Diabetes + OPLE 1000 mg kg**^**-1**^	
	**4 weeks**	**12 weeks**	**4 weeks**	**12 weeks**	**4 weeks**	**12 weeks**
Glomerulosclerotic index	0.23 ± 0.03	0.34 ± 0.03	1.14 ± 0.06^a^	1.36 ± 0.04^aa^	0.68 ± 0.06^a,b^	1.62 ± 0.03^aa^
Tubulointerstitial fibrosis index	0.49 ± 0.05	0.61 ± 0.06	2.48 ± 0.18^a^	2.75 ± 0.16^aa^	1.03 ± 0.08^b^	3.05 ± 0.07^aa^

Data are expressed as mean ± SEM of six experiments for each group. ^*a*^*P* < 0.05; ^*aa*^*P* < 0.01; vs. corresponding control; ^*b*^*P* < 0.05, vs. corresponding diabetes control.

Masson's trichrome-stained sections of diabetic kidney on week 4 exhibited increased collagen deposition, tubular dilation, and degeneration of cortical tubules (Figure 5iiiA) whilst these changes were not apparent in the C rat’s kidney. Marked tubulointerstitial fibrosis characterized by accumulation of extracellular matrix (ECM) protein in cortex and medulla was observed in diabetic kidney on week 12 (Figure 5iiiB). There was capillary occlusion, increased proliferation of interstitial fibroblasts, tubular dilatation and atrophy whereas no apparent changes were detected in kidney of C rats. Treatment with 1000 mg kg^−1^ OPLE for 4 weeks showed a reduction in tubulointerstitial fibrosis in comparison to DC rats (*P* < 0.05, Table 3). But diabetic animals treated with 1000 mg kg^−1^ OPLE for 12 weeks instead showed further increase in tubulointerstitial fibrosis, capillary occlusion, proliferation of interstitial fibroblasts, tubular dilatation and atrophy in comparison to DC rats, although morphometric analysis showed no significant difference (Figure 5iiiB, Table 3).

### NADPH oxidase subunit immunolocalisation and protein expression

**Figure 6:**
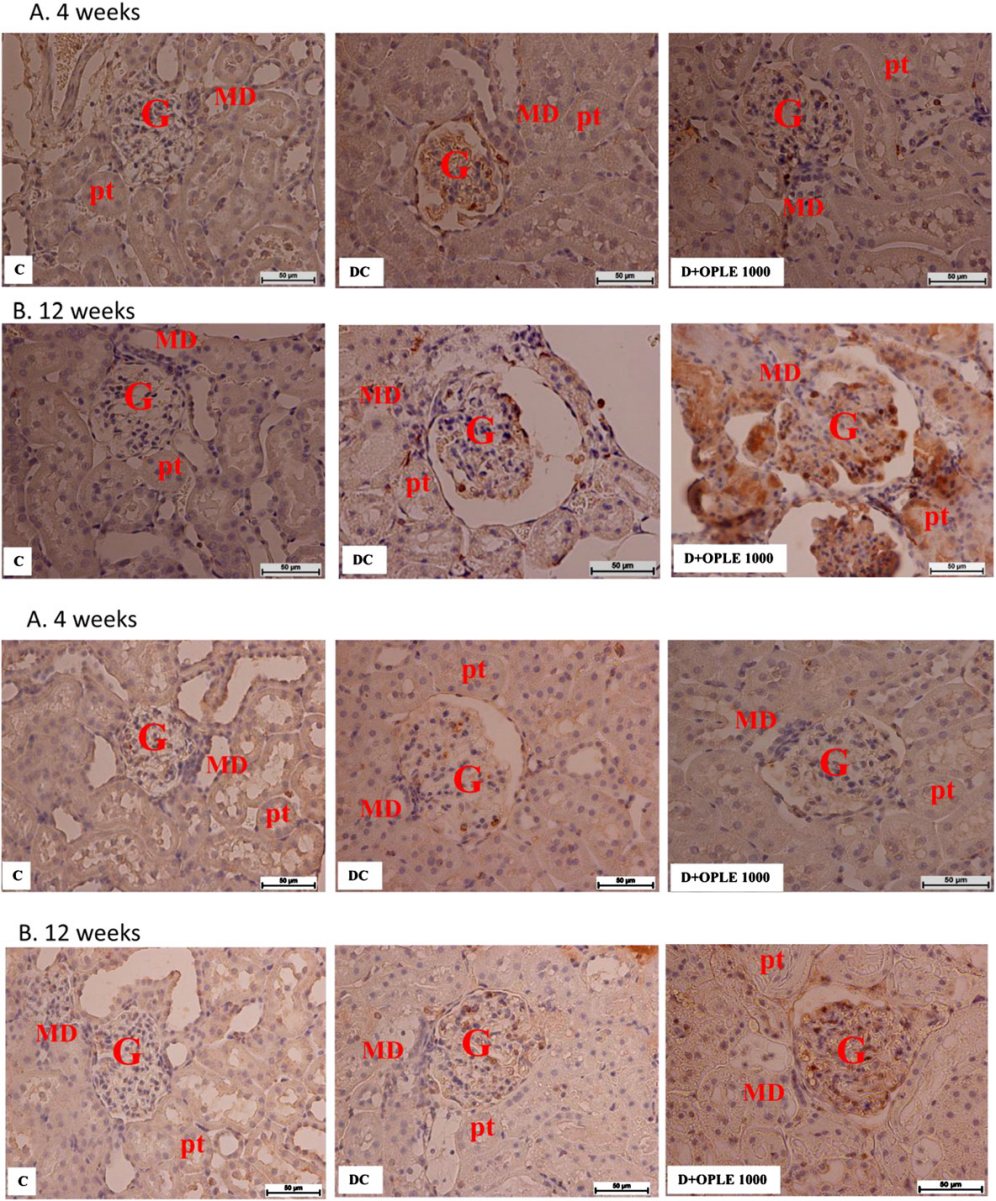
**NADPH oxidase subunits immunolocalization in the kidney.** Immunohistochemical staining of kidney section showing localization of nicotinamide adenine dinucleotide phosphate (NADPH) oxidase subunit (i) p22phox and (ii) p67phox in the 4-week **(A)** or 12-week **(B)** study. Control (C), Diabetes Control (DC) and Diabetes + OPLE 1000 mg kg^−1^ (D + OPLE 1000); MD, macula densa; G, glomerulus, pt, proximal tubule; Bar = 50 μm.

**Figure 7:**
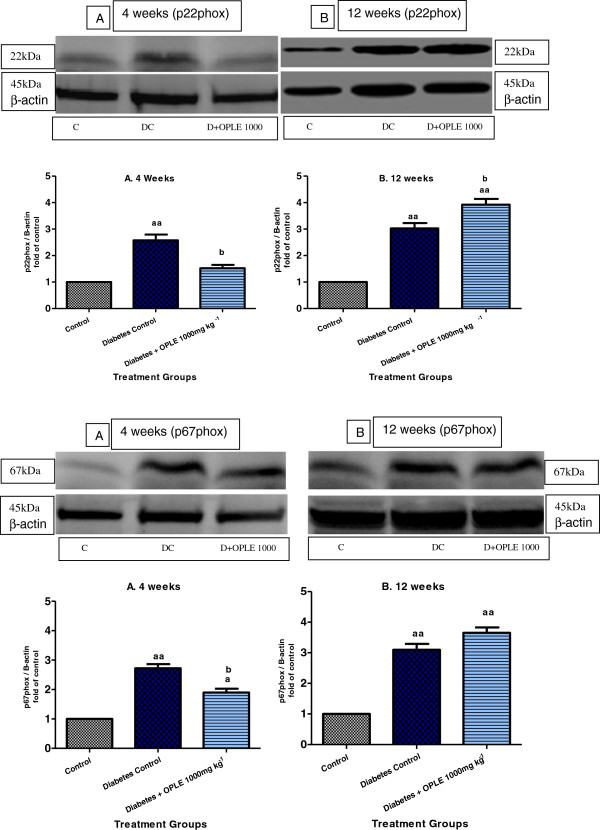
**NADPH oxidase subunits protein expression in the kidney.** Effect of OPLE on renal protein expressions of p22phox and p67phox NADPH oxidase subunit 4-week **(A)** or 12-week study **(B)**. (i and iii) Representative western blots showing bands of p22phox, p67phox and β-actin as an internal control. Control (C), Diabetes Control (DC) and Diabetes + OPLE 1000 mg kg^−1^ (D + OPLE 1000); (ii and iv) Densitometric analysis of protein renal expression of p22phox and p67phox NADPH oxidase subunits. Data are expressed as mean ± SEM of four experiments for each group. ^*a*^*P* < 0.05; ^*aa*^*P* < 0.01; vs. corresponding control; ^*b*^*P* < 0.05, vs. corresponding diabetes control.

To examine the localisation and expression of p22phox and p67phox proteins in the rat kidney, we carried out immunostaining analysis 4 and 12 weeks after onset of diabetes. Immunohistochemical staining of the NADPH oxidase subunits p22phox and p67phox revealed widespread staining in the glomeruli (Figures 6iA and iiA), which is similar in distribution to that of previously observed in the normal rat’s kidney [22]. The expression of glomerular p22phox and p67phox was increased with diabetes and ameliorated by treatment with 1000 mg kg-1 OPLE for 4 weeks but not by treatment with the extract for 12 weeks. Western blot analysis confirmed that p22phox and p67phox proteins were up-regulated significantly in diabetic kidney on the 4^th^ week (2.5 and 2.7 folds of C rats respectively, *P* < 0.01) and on the 12^th^ week (3 and 3.1 folds of C respectively, *P* < 0.01). Both the NADPH oxidase subunits elevation was reduced by 1000 mg kg^−1^ OPLE in diabetic kidneys on the 4^th^ week (*P* < 0.05, Figures 7iA-ivA). When treatment with the extract was extended to 12 weeks, both the NADPH oxidase subunits in the diabetic kidney were instead further increased, and the difference was significant only for the p22phox subunit (*P* < 0.05, Figure 7iB-ivB).

## Discussion

### The effects of OPLE in diabetic nephropathy (DN)

The present study was conducted to investigate the antioxidant and pro-oxidant effects of chronically administering high dose of OPLE (1000 mg kg^-1^) in an animal model of DN. Oxidative stress has been considered to be a general pathogenic factor of diabetic complications including nephropathy [3,4]. Our current data demonstrated that oral administration with high dose of catechins-rich OPLE (1000 mg kg^-1^) to STZ-induced diabetic rats for 4 weeks attenuated renal dysfunction (hyperfiltration, proteinuria) and development of glomerulosclerosis and tubulointerstitial fibrosis, features that are associated with DN [23]. Concomitant with the beneficial effects of OPLE, we observed a suppression of the increases in oxidative stress markers (8-OHdG, LPO) and the fibrotic cytokine, TGF-β1. Our data confirmed the previously reported findings that oral administration albeit with lower doses of OPLE (200 mg kg^-1^ and 500 mg kg^-1^) for 4 weeks similarly attenuated renal dysfunction and renal pathology associated with DN in the STZ-induced diabetic rat due to the antioxidant effect of the extract [5]. In contrast and most interestingly, our study revealed that when oral administration of 1000 mg kg^-1^ OPLE was extended for 12 weeks, no renoprotection was detected in the diabetic animals. In fact, worsening of renal dysfunction as evidenced by further increase in hyperfiltration and proteinuria were observed in these diabetic rats. Moreover, oxidative stress markers (LPO) and TGF-β1 were further elevated in diabetic rats treated with this dose of OPLE for 12 weeks as compared to the untreated diabetic rats possibly indicating pro-oxidant effect of OPLE. In corroboration, structural damage was amplified, although not significantly. It is noteworthy that our previous study with lower doses of OPLE (200 mg kg^-1^ and 500 mg kg^-1^) treated daily for the same duration (12 weeks) however provided optimal renoprotection in rats with DN in conjunction with suppression of the increases in oxidative stress markers [5].

### NADPH activity as a possible target of OPLE

In the present study, to explore the underlying molecular mechanisms with the aim of elucidating at least part of the antioxidant property of OPLE, we examined the effects of the extract on the renal expression of NADPH oxidase subunits, p22phox and p67phox. It is well established that oxidative stress has been implicated in the pathogenesis of renal injury in diabetes mellitus and the NADPH oxidase is an important source of ROS production [3,24,25]. The NADPH oxidase consists of membrane-bound subunits (p22phox and Nox4, a renal homologue of gp91phox) and cytosolic subunits (p47phox, p40phox, p67phox, and Rac) [26,27]. Previous studies suggested that one of the mechanisms contributing to increased oxidative stress in the diabetic kidney is increased expression of NADPH subunits, namely p22phox, p47phox, p67phox and Nox4 [1,22,28]. In consistent with these reports, we demonstrated through western blotting and immunohistochemistry that the renal expression of the membrane-bound subunit p22phox and the cytosolic subunit p67phox were enhanced in rats with 4 and 12 weeks diabetes respectively. A significant finding in this study was that administration of OPLE at 1000 mg kg^-1^ for 4 weeks reduced the diabetes-associated up-regulation of both subunits and this effect was independent of changes in blood sugar. We therefore hypothesise that inhibition of NADPH oxidase, the enzyme that is involved in formation of O_2_^.-^, may in part contribute to the antioxidant effect of OPLE. Studies have adduced evidence that catechins and their metabolites were capable of protecting vascular endothelial cells against O_2_^.-^ through inhibition of endothelial NADPH oxidase activity [29]. OPLE is rich in catechins such as epigallocatechin, catechin, epicatechin, epigallocatechin gallate and epicatechin gallate [10], and inhibition of NADPH oxidase in the present study in all probability could be due to the actions of these catechins, although other compounds such as ferulic acid which is also a component of OPLE [5], may also play a role. Indeed, ferulic acid has been shown to have higher NADPH oxidase-inhibitory potency than apocynin [29].

### Increase endogenous antioxidant by OPLE

The present study also demonstrated that OPLE enhanced endogenous antioxidant enzyme as one of the mechanisms to reduce diabetes-induced oxidative renal damage. Diabetic rats given 1000 mg kg^-1^ OPLE over a period of 4 weeks exhibited increased levels of GSH. Similarly, tea administration of which catechins are major components, prevented depletion in rats’ liver GSH induced by carbon tetrachloride or ethanol [30,31].

### Pro-oxidant property of OPLE

Our data clearly suggest that there is more than one mechanism that may contribute to the antioxidant effect of OPLE which could be beneficial in abrogating indices of DN. The beneficial effects of antioxidants mainly spotlight on their defensive functions against undue oxidative damage induced by ROS. However, from a health perspective one must be conscious that a powerful antioxidant could also exhibit pro-oxidant performance, leading to oxidative damage of cellular mechanism [16,32]. Indeed, what is intriguing in our present study was the unmasking of the pro-oxidant effect of OPLE when 1000 mg kg^-1^ of the extract was given to diabetic rats for an extended period i.e. 12 weeks as opposed to 4 weeks. We are not certain of the mechanisms that trigger the transition of OPLE antioxidant effect to the pro-oxidant effect when administered for a longer duration; however our findings showed increase expression of the NADPH subunits, p22phox (significant) and p67phox (non-significant) when 1000 mg kg^-1^ OPLE was administered to diabetic animals for 12 weeks. Aggravation of renal dysfunction and structural injury by the high dose of OPLE administered to diabetic animals for 12 weeks in the present study is purported to be due to the pro-oxidant effect of OPLE but we would not exclude some other unknown ways. Szeto et al. [33] reported that 200 μM epigallocatechin and epigallocatechin gallate induced oxidative damage in human DNA due to the production of hydrogen peroxide. Green tea extract (10–200 μg ml^-1^) which contain catechins similar to OPLE, and epigallocatechin gallate (20–200 μM) have also been shown to exacerbate oxidant activity, oxidative stress, genotoxicity and cytotoxicity induced by hydrogen peroxide in RAW 264.7 macrophages [34].

## Conclusion

The antioxidant/pro-oxidant properties of OPLE could be important in determining the functional outcome of a cell, and the biological response could either be beneficial or harmful, depending on the oxidative condition existing within a cell. Taken together, the findings of our study indicate that chronic administration of 1000 mg kg^-1^ OPLE exerts both antioxidant and pro-oxidant effects depending on the duration of treatment as assessed by levels of oxidative stress markers, renal dysfunction and renal pathology in DN. Furthermore, our study provides some mechanistic insight into the antioxidant and pro-oxidant effects of OPLE. Ultimately, our findings stress the importance of conducting a careful dose–response and treatment duration studies for OPLE before excessive intake of the product can be recommended to diseased individuals where oxidative stress plays a major role.

## Competing interests

The authors declare that they have no competing interests.

## Authors’ contributions

VR conducted all the studies and drafted the manuscript. IC gave advice on the conduct of the western blotting, analysis and interpretation of the data. MAA and NMK interpreted all the histological sections of the kidney. MZAS revised the manuscript critically for the important intellectual content. NAA was responsible for the conception and design of the study, drafted the manuscript and revised it critically for the important intellectual content. All authors read and approved the final manuscript.

## Pre-publication history

The pre-publication history for this paper can be accessed here:

http://www.biomedcentral.com/1472-6882/13/242/prepub

## References

[B1] Bhatti F Richard WM Laureano A Mark TQ William JW Christine M **Mechanisms of antioxidant and pro**-**oxidant effects of** α-**lipoic acid in the diabetic and nondiabetic kidney** Kidney Int 2005 67 1371 1380 10.1111/j.1523-1755.2005.00214.x 15780089

[B2] Nishikawa T Edelstein D Du XL Yanagishi S Matsumura T Kaneda Y Yorek MA Beebe D Oates PJ Hammes HP Giardino I Brownlee M Normalizing mitochondrial superoxide production blocks three pathways of hyperglycaemia damage Nature 2000 404 787 790 10.1038/35008121 10783895

[B3] Forbes JM Coughlan MT Cooper ME Oxidative stress as a major culprit in kidney disease in diabetes Diabetes 2008 57 6 1446 1454 10.2337/db08-0057 18511445

[B4] Giacco F Brownlee M Oxidative stress and diabetic complications Circ Res 2010 107 9 1058 1070 10.1161/CIRCRESAHA.110.223545 21030723 PMC2996922

[B5] Varatharajan R Munavvar ZAS Mahmood AA Normadiah MK Nor Azizan A Chronic administration of oil palm (*Elaeis guineensis*) leaves extract attenuates hyperglycaemic-induced oxidative stress and improves renal histopathology and function in experimental diabetes Evid Based Complement Alternat Meddoi:10.1155/2012/195367 10.1155/2012/195367 PMC3514844 23243433

[B6] Prabhakar S Starnes J Shi S Lonis B Tran R Diabetic nephropathy is associated with oxidative stress and decreased renal nitric oxide production J Am Soc Nephrol 2007 18 2945 2952 10.1681/ASN.2006080895 17928507

[B7] Chabrashvili T Tojo A Onozato ML Kitiyakara C Quinn MT Fujita T Welch WJ Wilcox CS Expression and cellular localization of classic NADPH subunits in the spontaneously hypertensive rat kidney Hypertension 2002 39 269 274 10.1161/hy0202.103264 11847196

[B8] Biswas SK Lopes de Faria JB Which comes first: renal inflammation or oxidative stress in spontaneously hypertensive rats? Free Radic Res 2007 41 216 224 10.1080/10715760601059672 17364948

[B9] Onozato ML Tojo A Goto A Fujita T Wilcox CS Oxidative stress and nitric oxide synthase in rat diabetic nephropathy: Effects of ACEI and ARB Kidney Int 2002 61 186 194 10.1046/j.1523-1755.2002.00123.x 11786100

[B10] Jaffri JM Mohamed S Ahmad IN Mustapha NM Manap YA Rohimi N Effects of catechin-rich oil palm leaf extract on normal and hypertensive rats kidney and liver Food Chem 2011 128 433 441 10.1016/j.foodchem.2011.03.050 25212153

[B11] Valko M Leibfritz D Moncol J Cronin MT Mazur M Telser J Free radicals and antioxidants in normal physiological functions and human disease Int J Biochem Cell Biol 2007 39 44 84 10.1016/j.biocel.2006.07.001 16978905

[B12] Pandey KB Rizvi SI Plant polyphenols as dietary antioxidants in human health and disease Oxid Med Cell Longev 2009 2 270 278 10.4161/oxim.2.5.9498 20716914 PMC2835915

[B13] Wajen W Michels G Steffan B Niering P Chovolou Y Kampkotter A Tran-Thi QH Proksch P Kahl R Low concentrations of flavonoids are protective in rat H4IIE cells whereas high concentration cause DNA damage and apoptosis J Nutr 2005 135 525 531 15735088 10.1093/jn/135.3.525

[B14] De Marchi U Biasutto L Garbisa S Toninello A Zoratti M Quercetin can act either as an inhibitor or an inducer of the mitochondrial permeability transition pore: A demonstration of the ambivalent redox character of polyphenols Biochim Biophys Acta 2009 1787 1425 1432 10.1016/j.bbabio.2009.06.002 19523917

[B15] Robaszkiewicz A Balcerczyk A Bartosz G Antioxidative and prooxidative effects of quercetin on A549 cells Cell Biol Int 2007 31 1245 1250 10.1016/j.cellbi.2007.04.009 17583542

[B16] Galati G Lin A Sultan AM O’Brien PJ Cellular and in vivo hepatotoxicity caused by green tea phenolic acids and catechins Free Radical Bio Med 2006 40 570 580 10.1016/j.freeradbiomed.2005.09.014 16458187

[B17] Doumas BT Bayse DD Borner K Carter RJ Elevitch F Garber CC Graby RA Hause LL Mather A Peters T Jr Rand RN Reeder DJ Russell SM Schaffer R Westgard JO A candidate reference method for determination of total protein in serum II. Test for transferability. Clin Chem 1981 27 1651 1654 7285315

[B18] Somogyi MA Method for the preparation of blood filtrates for the determination of sugar J Biol Chem 1930 86 655 663

[B19] Bojesen E A method for the determination of inulin in plasma and urine Acta Med Scand Suppl 1952 142 275 282 14902376 10.1111/j.0954-6820.1952.tb13376.x

[B20] Saito T Sumithran E Glasgow EF Atkins RC The enhancement of aminonucleoside nephrosis by the co-administration of protamine Kidney Int 1987 32 691 699 10.1038/ki.1987.262 3323599

[B21] Taneda S Pippin JW Sage EH Hudkins KL Takeuchi Y Couser WG Alpers CE Amelioration of DN in SPARC-null mice J Am Soc Nephrol 2003 14 968 980 10.1097/01.ASN.0000054498.83125.90 12660331

[B22] Etoh T Inoguchi T Kakimoto M Sonoda N Kobayashi K Kuroda J Sumimoto H Nawata H Increased expression of NAD(P)H oxidase subunits, NOX4 and p22phox, in the kidney of streptozotocin-induced diabetic rats and its reversibility by interventive insulin treatment Diabetologia 2003 46 1428 1437 10.1007/s00125-003-1205-6 13680125

[B23] Dronavalli S Duka I Bakris GL The pathogenesis of diabetic nephropathy Nat Clin Pract Endocrinol Metab 2008 4 8 444 452 10.1038/ncpendmet0894 18607402

[B24] Satoh M Fujimoto S Haruna Y Arakawa S Horike H Komai N Sasaki T Tsujioka K Makino H Kashihara N NAD(P)H oxidase and uncoupled nitric oxide synthase are major sources of glomerular superoxide in rats with experimental diabetic nephropathy Am J Physiol-Renal Physiol 2005 288 1144 1152 10.1152/ajprenal.00221.2004 15687247

[B25] Palsamy P Subramanian S Resveratrol protects diabetic kidney by attenuating hyperglycaemia-mediated oxidative stress and renal inflammatory cytokines via Nrf2-Keap1 signalling Biochim Biophys Acta 1812 2011 719 731 10.1016/j.bbadis.2011.03.008 21439372

[B26] Babior BM NADPH oxidase Curr Opin Immunol 2004 16 42 47 10.1016/j.coi.2003.12.001 14734109

[B27] Griendling KK Sorescu D Ushio-Fukai M NAD(P)H oxidase: Role in cardiovascular biology and disease Circ Res 2000 86 494 501 10.1161/01.RES.86.5.494 10720409

[B28] Kitada M Koya D Sugimoto T Isono M Araki S Kashiwagi A Haneda M Translocation of glomerular p47phox and 67phox by protein kinase C-beta activation is required for oxidative stress in diabetic nephropathy Diabetes 2003 52 2603 2614 10.2337/diabetes.52.10.2603 14514646

[B29] Steffen Y Gruber C Schewe T Sies H Mono-O-methylated flavanols and other flavonoids as inhibitors of endothelial NADPH oxidase Arch Biochem Biophys 2008 469 209 219 10.1016/j.abb.2007.10.012 17996190

[B30] Sur-Altiner D Yenice B Effect of black tea on lipid peroxidation in carbon tetrachloride treated male rats Drug Metabol Drug Interact 2000 16 123 128 10962644 10.1515/dmdi.2000.16.2.123

[B31] Skrzydiewska E Ostrowska J Farbiszewski R Michalak K Protective effect of green tea against lipid peroxidation in the rat liver, blood serum and the brain Phytomedicine 2002 9 232 238 10.1078/0944-7113-00119 12046864

[B32] Heim KE Tagliaferro AR Bobilya DJ Flavonoid antioxidants: chemistry, metabolism and structure-activity relationships J Nutr Biochem 2002 13 10 572 584 10.1016/S0955-2863(02)00208-5 12550068

[B33] Szeto YT Benzie IF Effects of dietary antioxidants on human DNA *ex vivo* Free Radic Res 2002 36 113 118 10.1080/10715760210161 11999698

[B34] Elbling L Weiss RM Teufelhofer O Uhl M Knasmueller S Schulte-Hermann R Berger W Micksche M Green tea extract (−)-epigallocatechin-3-gallate, the major tea catechins, exert oxidant but lack antioxidant activities FASEB J 2005 19 807 9 15738004 10.1096/fj.04-2915fje

